# Genome Sequence of a New “*Candidatus*” Phylum “Dependentiae” Isolate from Chiba, Japan

**DOI:** 10.1128/mra.01123-21

**Published:** 2022-02-03

**Authors:** Masaharu Takemura

**Affiliations:** a Laboratory of Biology, Institute of Arts and Sciences, Tokyo University of Science, Shinjuku, Tokyo, Japan; Indiana University, Bloomington

## Abstract

Little is known about the bacterial phylum “*Candidatus* Dependentiae,” because only three isolates have been reported. Here, I report the isolation and genome sequencing of a new member of this phylum, strain Noda2021. This is the fourth strain isolated from the phylum “*Candidatus* Dependentiae.”

## ANNOUNCEMENT

Knowledge of the candidate bacterial phylum “*Candidatus* Dependentiae” is limited, because only three isolates of this phylum have been reported to date (1–4), in addition to the metagenomic analysis (5). Here, I report a newly isolated strain, Noda2021, found in a pond in Noda City (Chiba Prefecture, Japan). To isolate the new microorganisms, I screened Vermamoeba vermiformis that had been infected with them.

First, a 5-mL sample of mud from a pond was mixed with 45 mL distilled water and incubated for 1.5 h at room temperature (20 to 24°C) with rotation, followed by incubation at 4°C for 1 h. After filtration through filter paper with a pore size of 20 μm (grade 43; Whatman International, Maidstone, UK), a 4.5-mL sample was mixed with 4.5 mL 2× proteose peptone-yeast extract-glucose (PYG) medium, 100 μL vermamoebae (3.0 × 10^5^ cells), and 360 μL antibiotic solution, which contained 100 μg mL^−1^ penicillin-streptomycin, 100 μg mL^−1^ ampicillin, and 5 μg mL^−1^ amphotericin B, as described previously (6). This mixture was added to a 96-well plate (100 μL per well).

**FIG 1:**
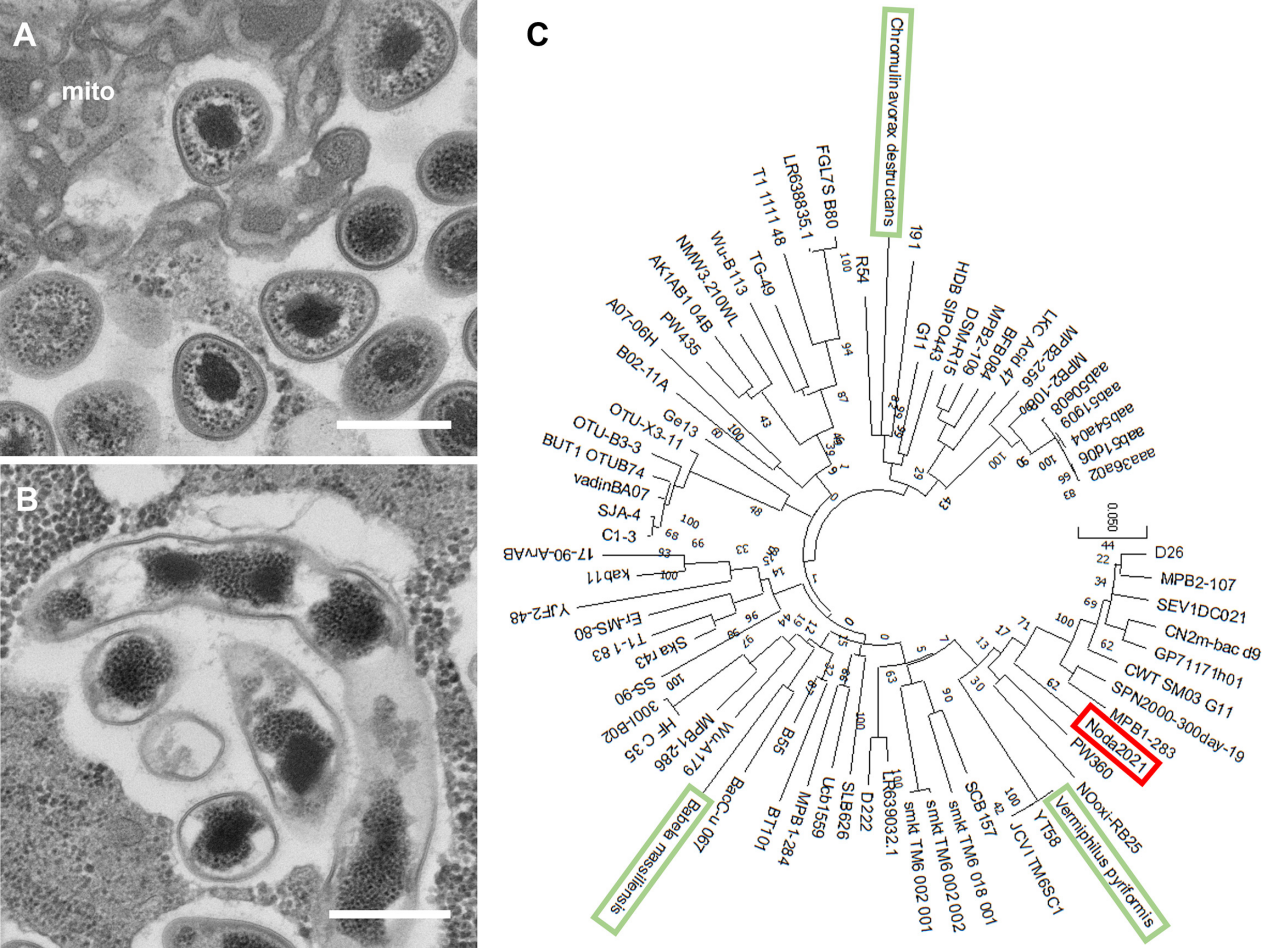
(A and B) Transmission electron microscopy (TEM) images of strain Noda2021 of the phylum “*Candidatus* Dependentiae.” Bar: 400 nm. Panel A indicates individual cells of Noda2021, surrounded by metamorphosed mitochondria (mito). Panel B indicates connected cells, probably before individualization, as previously reported in other isolates of the phylum “*Candidatus* Dependentiae” (4). (C) Phylogenetic tree of the 16S rRNA sequences. The analysis was performed using the maximum likelihood method with 1,000 replicates using MEGA X software (7). The red box indicates the new isolate in this study. The green boxes indicate previously isolated strains.

After 5 days of culture at 26°C, 10 μL supernatant from each well showing microscopic evidence of cytopathic effects (CPE; cell rounding or fusiform morphology) was serially diluted 10^11^-fold with PYG. Then, 10 μL each dilution was mixed with 90 μL PYG medium containing 9.0 × 10^4^ vermamoeba cells in a 96-well plate. After 5 days, fresh vermamoeba cells in a 12-well plate were inoculated with the highest dilution of supernatants exhibiting CPE, followed by inoculation in a 25-cm^2^ culture flask several days later. The supernatant of the vermamoeba cell culture exhibiting CPE was then harvested. Cells of this organism, which infect and proliferate in vermamoebae, were collected by centrifugation at 8,000 × *g* for 35 min at 4°C from the supernatant of the infected vermamoeba culture. The resulting pellets were resuspended and washed with 1 mL phosphate-buffered saline (PBS) prior to use for DNA extraction. For imaging, *V. vermiformis* cells were cultured in PYG medium in 75-cm^2^ culture flasks and infected with strain Noda2021. Two days after infection, *V. vermiformis* cells were collected by centrifugation at 500 × *g* for 5 min. The cells were washed with PBS, followed by fixation with 2% glutaraldehyde (GA) in PBS and staining with 2% osmium tetroxide. The cells were then dehydrated in ethanol solutions of increasing concentration and embedded in Epon 812. Ultra-thin sections were prepared and stained with 2% uranyl acetate (6). Transmission electron microscopy (TEM) was performed using a model JEM-1400 microscope (JEOL Ltd., Tokyo, Japan) at the Hanaichi UltraStructure Research Institute (Aichi, Japan) (Fig. 1A and B).

Genomic DNA (gDNA) was extracted from this organism using the NucleoSpin tissue XS kit (Macherey-Nagel GmbH & Co. KG, Duren, Germany) according to the manufacturer’s instructions. Sequencing was performed using an RS II device (PacBio, Menlo Park, CA, USA). For PacBio RS II sequencing, 8 g genomic DNA was used for preparation of a 20-kb library. For gDNA where the size range was less than 17 kb, I used the Bioanalyzer 2100 (Agilent) to determine the actual size distribution. The gDNA was sheared using a g-TUBE device (Covaris Inc., Woburn, MA, USA) and purified using AMPure PB magnetic beads (Beckman Coulter Inc., Brea, CA, USA) if the apparent size was greater than 40 kb. A library with a total size of 10 μL was prepared using the PacBio DNA template prep kit v1.0 (for 3 to 10 kb). The PacBio DNA sequencing kit v4.0 and 8 single-molecule real-time (SMRT) cells were used for sequencing using the PacBio RS II sequencing platform. HGAP v3.0 software was used to assemble 131,940 subreads (1,251,501,591 subread bases) into one contig with a length of 1,222,284 nucleotides (*N*_50_ value, 13,552 bp). The GC content of the genome was 38.30%. Gene annotation was performed using the DFAST annotation tool on DDBJ (DNA Data Bank of Japan) sites. I identified 1,287 open reading frames (ORFs), 38 tRNAs, and 3 rRNAs (Fig. 1C).

### Data availability.

The sequence data are available at GenBank (accession number AP025250) under BioProject accession number PRJDB12443 and BioSample accession number SAMD00414974, and the raw reads can be found in the Sequence Read Archive (accession number DRR327641).
